# 296 Factors Associated with Receiving First-Line Antibiotics for Urinary Tract Infections in Urgent Care Clinics

**DOI:** 10.1017/ash.2026.10654

**Published:** 2026-06-23

**Authors:** Debby Ben David, Yael Cohen, Eduard Mazo, Julia Belichkov, Lili Raviv, Alona Haleva, Malka Yokobov, Maya Heled Akiva, Orna Schwartz

**Affiliations:** 1 Wolfson Medical Center; 2 wolfson medical center

## Abstract

**Background:** Active surveillance for carbapenem-resistant Acinetobacter baumannii (CRAB) is essential for infection prevention in endemic settings. Skin screening using pre-moistened sponges with enrichment has demonstrated the highest sensitivity for CRAB detection and is considered the reference standard. However, sponge-based sampling is labor-intensive and less feasible for routine use. Prior studies reported limited sensitivity of swab-based screening. We evaluated whether an optimized swab-based protocol, incorporating expanded skin sampling sites, could improve sensitivity compared with sponge sampling among known CRAB carriers. **Methods:** This paired-method study included hospitalized patients with documented prior CRAB carriage withing prior 3 months. On the day of screening, skin samples were collected before bathing simultaneously using Polywipe sponges and dry E-Swabs (Copan Italia S.P.A., Brescia, Italy), from opposite sides of the body. Swabs were used to sample multiple skin sites, including palm, interdigital spaces, antecubital fossa, axilla, groin, knee crease, sole of the foot, and toe webs, encompassing moist skin folds known to favor Acinetobacter colonization. Skin samples were collected from patients’ arms and legs (groin and downward), Sponge samples were incubated overnight in brain–heart infusion (BHI) broth at 37 °C, vortexed, and plated onto CHROMagar MDR Acinetobacter. Swabs were broken directly into 3 mL BHI broth, incubated overnight, vortexed, and plated identically. Sponge sampling was defined as the gold standard. Sensitivity of swab screening was calculated among sponge-positive samples. **Results:** Among 134 paired screening samples from known CRAB carriers, 95 (70.9%) were positive by sponge sampling. Of these, 78 were also positive by swab screening, yielding a swab sensitivity of 82.1% (95% CI, 73.2–88.5). Thirty-nine patients (29.1% of the cohort) screened negative by both methods on the day of sampling, despite prior documented carriage. Discordant results were predominantly sponge-positive/swab-negative. **Conclusions:** Among patients with known CRAB carriage, an optimized swab-based skin screening protocol achieved substantially higher sensitivity than previously reported swab methods, though sensitivity remained lower than sponge-based screening. The inclusion of moist skin folds (such as the knee creases and toe webs), which are known to harbor Acinetobacter spp. but are not routinely sampled in previous studies, may explain the improved detection observed. The high proportion of negative screeing among known carriers highlights the dynamic and intermittent nature of CRAB skin colonization and the limitations of single time-point screening.

